# Unsupervised clustering of PET/CT features in fever of unknown origin (FUO) and inflammation of unknown origin (IUO)

**DOI:** 10.3389/fmed.2026.1830800

**Published:** 2026-05-29

**Authors:** Sule Ceylan, Bahadır Ceylan, Oktay Olmuscelik, Tansel Cakir

**Affiliations:** 1 Department of Nuclear Medicine, University of Health Science Gaziosmanpasa Training and Research Hospital, Istanbul, Türkiye; 2 Department of Infectious Diseases and Clinical Microbiology, Medical Faculty, Istanbul Medipol University, Istanbul, Türkiye; 3 Department of Internal Medicine, Medical Faculty, Istanbul Medipol University, Istanbul, Türkiye; 4 Department of Nuclear Medicine, Medical Faculty, Istanbul Medipol University, Istanbul, Türkiye

**Keywords:** FUO, machine learning, PET/CT, prediction, unsupervised learning

## Abstract

**Aim:**

The aim of this study was to classify fever of unknown origin (FUO) patients based on PET/CT imaging features using unsupervised clustering, and to explore whether these patterns could provide insights into underlying etiologies.

**Materials and methods:**

We conducted a retrospective cohort study of adult FUO patients who underwent 18F-FDG PET/CT. For each patient, data were extracted on involvement of reticuloendothelial organs, parenchymal organs, and large vessels, including the maximum SUVmax within each involved system and the uptake pattern (focal, diffuse, or mixed). Patients were clustered based on these PET/CT features using four unsupervised algorithms (hierarchical clustering, hierarchical density-based spatial clustering of applications with noise, spectral clustering, K-prototypes clustering), and a consensus clustering approach was applied to derive robust final clusters. Cluster assignments were compared with final clinical diagnoses using the Adjusted Rand Index (ARI) and Mutual Information (MI) to explore clinical relevance.

**Results:**

The study included 289 FUO patients with a mean age of 59 ± 17 years, comprising 128 (44.3%) women and 161 (55.7%) men. Consensus clustering of PET/CT features in FUO patients identified four distinct clusters: Cluster 0 with combined reticuloendothelial, parenchymal, and large artery involvement, Cluster 1 with prominent reticuloendothelial and parenchymal involvement, Cluster 2 with mild reticuloendothelial involvement, and Cluster 3 with selective parenchymal involvement. Infections were the predominant etiologic category across all clusters, while rheumatologic diseases also contributed substantially, particularly in Cluster 2. Comparison with final diagnoses showed low concordance (ARI 0.006, NMI 0.047; Chi-square 32.46, *p* = 0.0012, Cramér’s V = 0.19), indicating that consensus clusters capture PET/CT phenotypes rather than specific etiologies.

**Conclusion:**

Consensus clustering of PET/CT features in FUO patients revealed distinct imaging phenotypes, but these clusters were not effective in directly identifying final clinical diagnoses, underscoring PET/CT’s complementary role in evaluation.

## 1 Introduction

Fever of unknown origin (FUO) represents a persistent diagnostic challenge because of its heterogeneous etiology, which includes infections, malignancies, autoimmune disorders, and other inflammatory conditions (1). Accurate and timely identification of the underlying cause is crucial for guiding targeted therapy and improving patient outcomes (1). However, conventional diagnostic approaches—such as laboratory tests, imaging studies, and tissue biopsies—may fail to establish a definitive diagnosis in patients with complex and non-specific clinical presentations (1).

In recent years, Fluorine-18 Fluorodeoxyglucose Positron Emission Tomography / Computed Tomography (PET/CT) has gained an increasingly important role in the diagnostic evaluation of FUO, regardless of whether the underlying cause is infectious, malignant, or inflammatory (2–10). Most studies in the literature have assessed the usefulness of PET/CT by categorizing cases as true positive or true negative (considered clinically useful) and false positive or false negative (considered not useful), and have primarily focused on reporting the overall diagnostic utility of PET/CT in FUO (2–9). However, relatively few studies have attempted to directly predict the final diagnosis using intrinsic PET/CT imaging features, such as SUVmax and anatomical distribution of uptake (11–14). Moreover, in these studies, the classification of anatomical uptake locations has generally been limited, and uptake patterns have often not been systematically incorporated into the analysis. In addition, most prior investigations have relied primarily on conventional statistical methods, while the use of machine learning approaches in this context remains scarce.

Therefore, considering these limitations in the literature, we designed a study with a different methodological framework to better evaluate the diagnostic role of PET/CT in FUO.

In this study, we aimed to classify FUO patients solely based on mixed numerical and categorical PET/CT features using multiple unsupervised clustering algorithms. We then evaluated whether these PET/CT-derived patterns could predict the final clinical diagnosis, including infection, rheumatologic disease, malignancy, or other conditions. This approach is novel because it assesses PET/CT imaging features directly as potential predictive markers, rather than relying solely on the *post hoc* confirmation of focal lesions.

## 2 Materials and methods

### 2.1 Study population and data extraction

This study is a retrospective cohort study. Patients with FUO and inflammation of unknown origin (IUO) who were hospitalized or followed as outpatients at Istanbul Medipol University between 2016 and 2026 were included in the study. Ethical approval for the study was obtained from the Ethics Committee of Istanbul Medipol University.

#### 2.1.1 Inclusion criteria

- Age ≥18 years.
- Diagnosis of FUO or IUO.
- PET/CT performed during the evaluation of FUO or IUO.

#### 2.1.2 Exclusion criteria

- No PET/CT performed.
- Patients lost to follow-up before final diagnosis was established.
- Age <18 years.

The following PET/CT variables were included for clustering analysis: involvement of reticuloendothelial organs, involvement of parenchymal organs, involvement of large vessels, number of reticuloendothelial organs involved, number of parenchymal organs involved, number of large arteries involved, maximum SUVmax of lymphoreticular system involvement, maximum SUVmax of parenchymal organ involvement, maximum SUVmax of large artery involvement, and the pattern of involvement for each compartment. Reticuloendothelial organs were defined as lymph nodes, spleen, and bone marrow. Large vessel involvement included the aorta, subclavian arteries, carotid arteries, brachiocephalic artery, femoral arteries, and iliac arteries. Patterns of involvement were classified as focal, diffuse, or mixed (diffuse with superimposed focal lesions).

### 2.2 PET/CT protocol

All patients underwent whole-body 18F-FDG PET/CT imaging using a Siemens Biograph mCT scanner. Prior to tracer injection, patients fasted for at least six hours, and blood glucose levels were confirmed to be below 150 mg/dL. An intravenous dose of 0.1 mCi/kg (3.7 MBq/kg) of 18F-FDG was administered, followed by a 60-min uptake period in a quiet, dimly lit room to minimize muscular activity and physiologic uptake. The scan extended from the skull base to the mid-thigh, with patients positioned supine and arms raised whenever feasible. A low-dose CT was first acquired for attenuation correction and anatomical localization (120 kV, 80–200 mAs, pitch 0.8–1.0, slice thickness 3–5 mm), followed by PET emission imaging at 2–3 min per bed position in 3D acquisition mode. Images were reconstructed using an OSEM algorithm with corrections for attenuation, scatter, random coincidences, and radioactive decay. Fused PET/CT images were reviewed on a dedicated Siemens syngo.via workstation.

All PET/CT scans were retrospectively reviewed by a single experienced nuclear medicine physician. The reader was blinded to the final clinical diagnosis at the time of image interpretation.

### 2.3 Clustering analyses

Patients were clustered solely based on PET/CT features using five clustering approaches to explore potential structural patterns within the data:

a) Hierarchical clustering
b) HDBSCAN (Hierarchical Density-Based Spatial Clustering of Applications with Noise)
c) Spectral clustering
d) K-prototypes clustering.
e) Consensus clustering.

For hierarchical clustering, HDBSCAN, and spectral clustering, pairwise dissimilarities were calculated using the Gower distance to account for mixed-type variables, and the resulting distance matrix was used as input. For K-prototypes clustering, continuous variables were standardized and categorical variables numerically encoded prior to analysis, allowing direct clustering on the processed dataset.

The Gower distance matrix was visualized as a heatmap to explore patterns of similarity and dissimilarity among patients. To assess the intrinsic clustering tendency of the dataset based on the Gower distance matrix prior to clustering, the Hopkins statistic was computed, providing a quantitative measure of the dataset’s clusterability.

#### 2.3.1 Hierarchical clustering

Hierarchical agglomerative clustering was performed using the Gower distance matrix. The resulting dendrogram was visually inspected to identify a level at which a substantial increase in linkage distance occurred, indicating a natural separation between patient groups. The dendrogram was then cut at this threshold to define the final clusters, with each branch below the cut corresponding to a distinct cluster.

#### 2.3.2 HDBSCAN

For HDBSCAN, multiple combinations of min_cluster_size (5, 10, 15–17) and min_samples (set to none, 5, or 10) were systematically evaluated. The final configuration was selected based on cluster stability (cluster persistence), the number of clinically meaningful clusters obtained, and an acceptable proportion of noise points. Using these optimized parameters, HDBSCAN was applied to the Gower distance matrix to identify patient clusters.

#### 2.3.3 Spectral clustering

Spectral clustering was applied to explore patterns in PET-derived FUO patient data. The similarity matrix was defined as 1 minus the Gower distance to account for mixed-type variables, and the normalized Laplacian of this similarity matrix was computed.

To determine the optimal number of clusters, two complementary approaches were used. For the eigengap method, the first 15 eigenvalues of the normalized Laplacian were examined, and notable increases between consecutive eigenvalues were interpreted as indications of meaningful separations in the data. For the silhouette analysis, spectral clustering was performed for cluster numbers ranging from 2 to 8, and silhouette scores were computed to assess cluster compactness and separation. These methods guided the selection of the most appropriate number of clusters for spectral clustering analyses. Once the optimal number of clusters was identified, spectral clustering was applied to assign patients to groups accordingly.

#### 2.3.4 K-prototypes clustering

To identify the optimal number of clusters, K-Prototypes clustering was performed for cluster numbers ranging from 2 to 8. The within-cluster dissimilarity (cost) was computed for each k, and the rate of decrease in cost was assessed using the elbow method, which identifies the point beyond which increasing the number of clusters yields minimal improvement in clustering quality. Based on this assessment, the most appropriate number of clusters was selected, providing a basis for subsequent stratification and analyses. Using this optimal cluster number, K-Prototypes clustering was then applied to assign patients to clusters for subsequent analyses.

### 2.4 Consensus clustering

To derive a robust final clustering solution, results from the four clustering algorithms (Hierarchical clustering, HDBSCAN, spectral clustering and, k-prototypes clustering) were integrated using a consensus clustering approach. For each patient, cluster assignments obtained from hierarchical clustering, HDBSCAN, spectral clustering, and K-prototypes clustering were combined. The final cluster label was determined using a majority voting strategy across methods, resulting in a consensus cluster assignment for each patient. These consensus clusters were used for subsequent analyses.

### 2.5 Characterization of clusters

For each cluster, the number of organs involved, the patterns of involvement, and the maximum SUVmax values were determined for the lymphoreticular system, large arteries, and parenchymal organs, and the distribution of final diagnoses within each cluster was also calculated.

### 2.6 Comparison of PET/CT features across consensus clusters

Subsequently, differences in PET/CT features across the resulting consensus clusters were examined using two complementary approaches. First, continuous PET/CT features (e.g., number of organs involved, maximum SUVmax) were compared across consensus clusters using the Kruskal-Wallis H test, a non-parametric method suitable for comparing multiple independent groups. For features with a significant Kruskal-Wallis *p*-value (*p* < 0.05), Dunn’s post-hoc test with Bonferroni adjustment was applied to identify which pairs of clusters differed significantly. Categorical features, including organ involvement patterns (integer-coded), were compared using the chi-square test of independence to evaluate overall differences between clusters. For features with a significant chi-square test, pairwise cluster comparisons were performed using chi-square tests with Bonferroni correction to adjust for multiple comparisons. A *p*-value < 0.05 was considered statistically significant for all tests after adjustment for multiple comparisons.

Second, to quantify the magnitude of differences in continuous PET/CT features between consensus clusters, standardized mean differences (SMD) were calculated. The SMD is defined as the difference between two cluster means divided by their pooled standard deviation, providing an effect size measure that is independent of the sample size:

$\mathrm{SMD}=(\bar{X}₁—\bar{X}₂)/\text{sqrt}(((n₁-1)\cdot s{₁}^{2}+(n₂-1)\cdot s{₂}^{2})/(n₁+n₂—2))$

Where:

- X̄₁, X̄₂ are the means of the two clusters.
- s₁, s₂ are the standard deviations.
- n₁, n₂ are the sample sizes.

SMDs were calculated pairwise for all cluster comparisons. A positive SMD indicates that the feature mean is higher in the first cluster, while a negative SMD indicates it is higher in the second cluster.

### 2.7 UMAP visualization of clustering results

To visually represent the clustering structure of PET-derived FUO patient data, UMAP (Uniform Manifold Approximation and Projection) was applied for dimensionality reduction. The high-dimensional PET/CT features were projected into two dimensions (UMAP1 and UMAP2) to preserve both local and global relationships among patients.

Clustering results from hierarchical clustering, HDBSCAN, spectral clustering, consensus clustering, and K-Prototypes clustering were overlaid on the UMAP coordinates, with each cluster assigned a distinct color. Noise points identified by HDBSCAN were represented in gray. This visualization allowed for intuitive comparison of cluster assignments across different algorithms and facilitated interpretation of patient grouping patterns in a two-dimensional embedding space.

### 2.8 Cluster stability assessment

Cluster stability was evaluated using bootstrap resampling. For each method (Hierarchical, Spectral, HDBSCAN, K-Prototypes, Consensus), 50 bootstrap samples were generated by resampling rows with replacement. Clustering was repeated on each sample, and the similarity to the original clustering was quantified using the Adjusted Rand Index (ARI). Mean and standard deviation of ARI scores were used to summarize stability.

### 2.9 Cluster validation and comparison with final diagnoses

The resulting cluster assignments from each method were compared with final clinical diagnoses to evaluate their clinical relevance. Associations were assessed using the chi-square test and Cramér’s V coefficient. Agreement between clustering results and diagnostic categories was further evaluated using the ARI and Normalized Mutual Information (NMI), providing complementary measures of similarity between the clustering assignments and the reference diagnoses.

### 2.10 Comparative analyses between FUO and IUO patients

Since the study population consisted of both FUO and IUO patients, additional subgroup analyses were performed to investigate potential differences between these two groups. Differences in final etiologic diagnoses between FUO and IUO patients were evaluated using the chi-square test. In the univariate analysis, chi-square tests were also used to assess differences in PET/CT involvement patterns between the two groups. Variables found to be significant in univariate analyses were subsequently included in the multivariate logistic regression analysis. A *p* value <0.05 was considered statistically significant in all analyses. All statistical analyses were performed using IBM SPSS Statistics for Windows, Version 2025 (IBM Corp., Armonk, NY, United States).

## 3 Result

All variables included in the clustering analysis were complete, and no missing data handling or imputation was required. A total of 289 patients who underwent PET/CT evaluation were included in our study. Among them, 128 (44.3%) were diagnosed with infections, 23 (8%) with malignancies, 68 (23.5%) with rheumatologic diseases, and 31 (10.7%) with other unclassified causes. This distribution indicates that infections are the most common etiology in FUO patients, while rheumatologic and malignant causes also represent significant subgroups. Of the patients, 174 (60.2%) had FUO, whereas 115 (39.8%) had IUO. No significant difference was observed in the etiologic distribution between FUO and IUO patients (Supplementary Table S1) (*p* = 192). Univariate chi-square analyses followed by multivariate logistic regression analyses were performed to evaluate differences in PET/CT involvement sites between FUO and IUO patients. Compared with IUO patients, FUO patients demonstrated more frequent large artery involvement and less frequent parenchymal involvement (Supplementary Table S2).

Based on PET-CT findings, the Gower distance between patients was calculated, and the resulting distance matrix was visualized as a heatmap (Supplementary Figure S1). The heatmap illustrates the similarity and dissimilarity among patients, revealing a heterogeneous structure in the dataset without any distinct clustering. The heatmap of the Gower distance matrix and the Hopkins statistic (*H* = 0.37) indicate a heterogeneous dataset without strong natural clustering, suggesting careful interpretation of subsequent cluster analysis.

Clustering of patients was performed using hierarchical clustering, HDBSCAN, and spectral clustering based on Gower distances to account for mixed-type PET/CT features. For K-prototypes clustering, the PET/CT features were used directly without distance transformation. The results obtained using all four clustering algorithms are summarized below.

### 3.1 Hierarchical clustering

Hierarchical clustering based on Gower distance was applied to PET/CT findings of FUO patients, and the resulting dendrogram revealed 4 distinct clusters (Supplementary Figure S2).

The differences in PET-CT findings among the four clusters obtained by applying the Gower distance to hierarchical clustering are presented in Supplementary Table S3.

Overall, the UMAP representation of PET/CT-derived features highlights the heterogeneity of FUO patient phenotypes, with some clusters clearly distinct while others exhibit overlapping characteristics (Supplementary Figure S3).

The distribution of final diagnoses in relation to the clusters generated using Gower distance followed by hierarchical clustering is presented in Supplementary Table S4.

### 3.2 HDBSCAN

To determine the optimal HDBSCAN parameters, multiple combinations of min_cluster_size (5, 10, 15–17) and min_samples (set to none, 5, 10) were systematically evaluated using the precomputed Gower distance matrix.

The final model parameters (min_cluster_size = 20, min_samples = 5) were selected based on cluster stability (cluster persistence), number of meaningful clusters obtained, and acceptable noise ratio. This configuration provided the most stable and interpretable clustering solution.

Application of HDBSCAN clustering using the Gower distance matrix identified five distinct clusters; the PET-CT features defining these clusters are summarized in Supplementary Table S5.

The clusters obtained using HDBSCAN are illustrated in a schematic UMAP plot generated from PET/CT-derived features (Supplementary Figure S4), highlighting the distinct separation between groups.

The relationship between the clusters obtained by applying HDBSCAN to the Gower distance matrix and the final diagnoses is presented in Supplementary Table S6.

### 3.3 Spectral clustering

**Figure 1:**
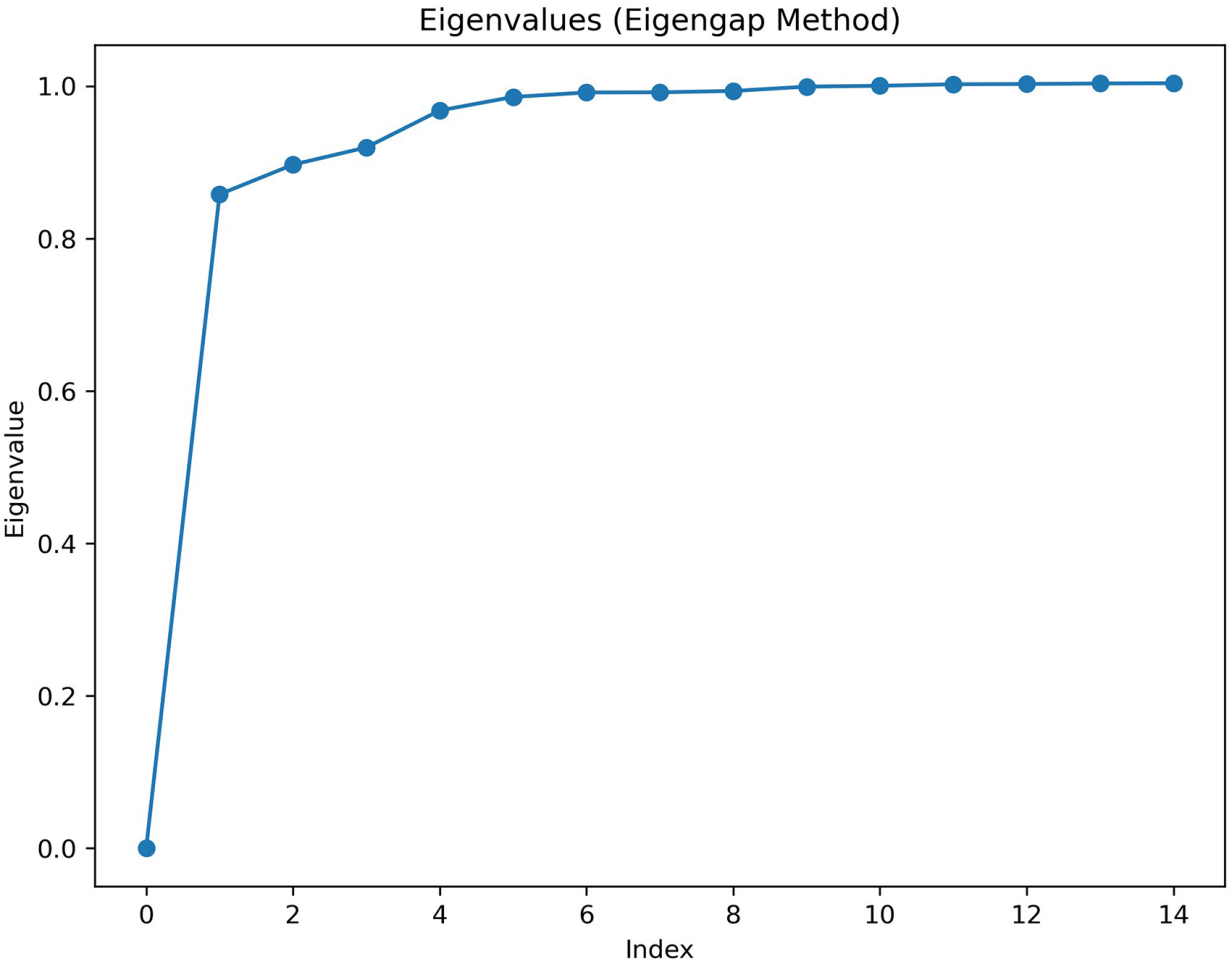
Eigenvalue plot used to estimate the optimal number of clusters. The clear gap supports the selection of four clusters.

Spectral clustering was applied to the PET-derived FUO patient data to uncover distinct patterns. To determine the optimal number of clusters, we first computed the eigenvalues of the normalized Laplacian of the similarity matrix (1 − Gower distance) and plotted the first 15 eigenvalues to assess the eigengap (Figure 1).

The plot revealed a notable gap after the fourth eigenvalue, suggesting that four clusters capture the most significant structure in the data. To further validate this choice, silhouette scores were computed for cluster numbers ranging from 2 to 8, evaluating cluster compactness and separation. The maximum silhouette score (0.514) was observed with four clusters, providing additional support for a four-cluster solution. Together, the eigengap and silhouette analyses indicate that dividing patients into four spectral clusters is reasonable and likely reflects meaningful phenotypic groupings based on PET features.

The PET-CT features characterizing the four clusters obtained by spectral clustering are summarized in Supplementary Table S7.

The four clusters identified through spectral clustering are illustrated in a two-dimensional UMAP plot generated from PET/CT-derived features (Supplementary Figure S5), highlighting the clear separation between groups.

The relationship between the clusters obtained by spectral clustering and the final diagnoses is summarized in Supplementary Table S8.

### 3.4 K-prototypes clustering

**Figure 2:**
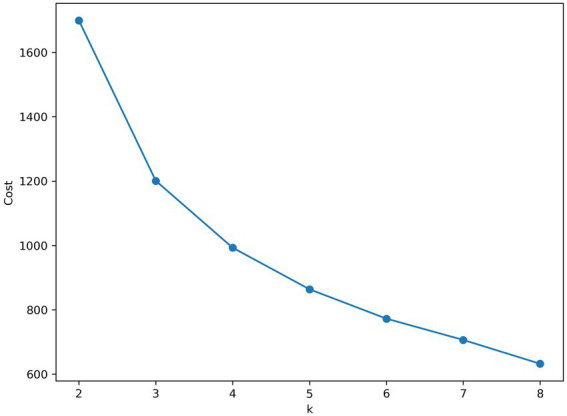
Elbow plot showing clustering performance across different numbers of clusters (*k* = 2–8). The curve flattens after *k* = 4, supporting the use of four clusters.

To determine the optimal number of clusters for the FUO patient dataset, K-Prototypes clustering was performed for *k* = 2 to *k* = 8 clusters. The cost (sum of within-cluster dissimilarities) decreased as k increased, as shown in the plotted curve (Figure 2).

The reduction in cost slowed noticeably after *k* = 4, indicating diminishing returns in clustering quality for higher k values. Based on this “elbow” method, four clusters were selected as the optimal number, balancing cluster separation and parsimony. This selection provided a meaningful stratification of patients for subsequent analyses, reflecting distinct phenotypic patterns.

The PET-CT features characterizing the clusters obtained from K-Prototypes analysis are presented in Supplementary Table S9.

The clusters identified through K-prototypes clustering are illustrated in a UMAP plot generated from PET/CT-derived features (Supplementary Figure S6), highlighting the clear separation between groups.

The distribution of final diagnoses across clusters derived from the K-prototypes clustering is shown in Supplementary Table S10.

After generating all clustering algorithms, the stability of the identified clusters was assessed using the bootstrap method.

### 3.5 Cluster stability assesment

**Figure 3:**
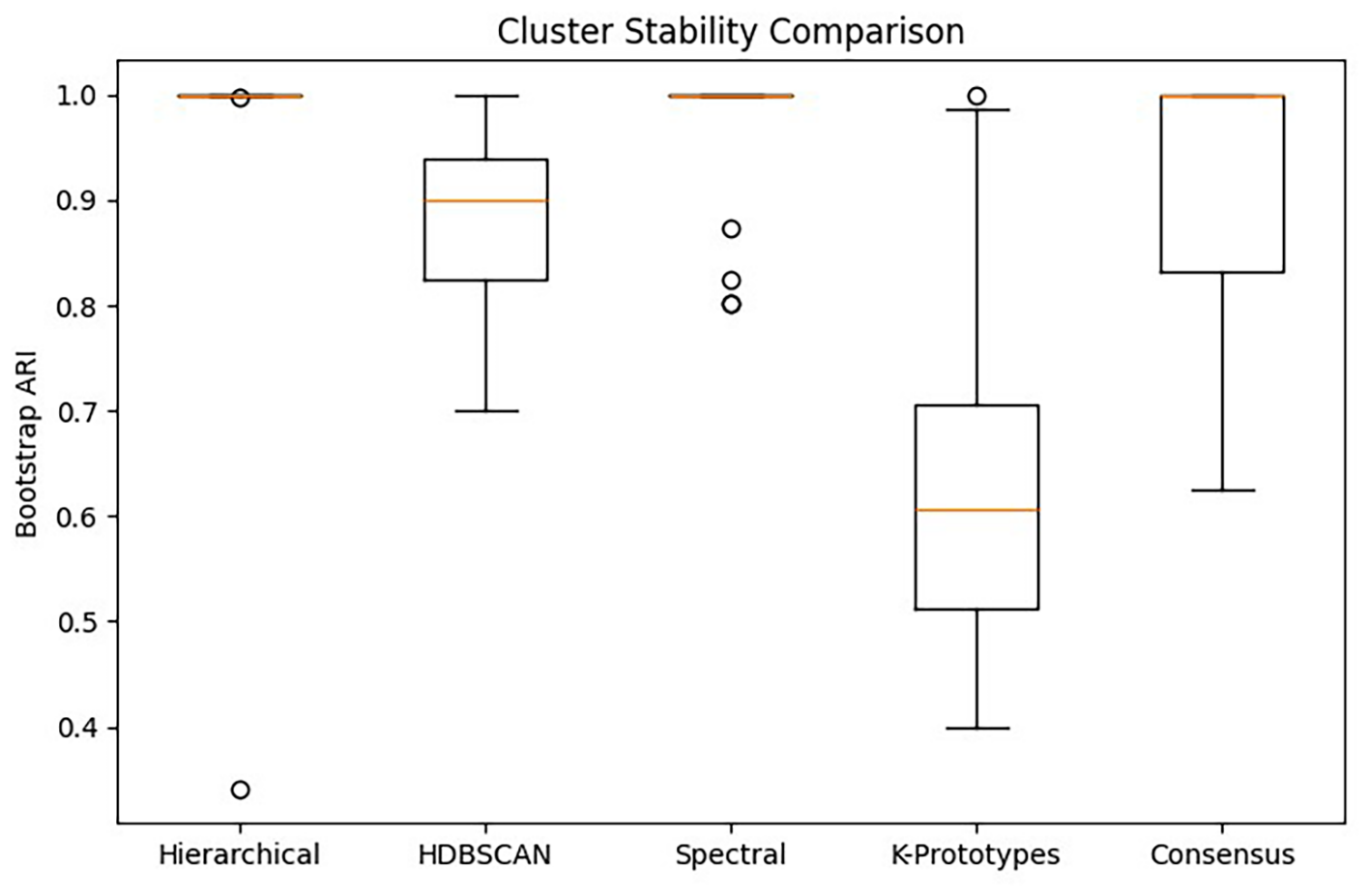
Comparison of clustering stability across different methods based on repeated sampling. Higher values indicate more stable clustering.

Clustering stability was evaluated using bootstrap resampling and the ARI (Figure 3, Table 1).

**Table 1:** Bootstrap analysis of clustering stability.

Method	Mean_ARI	SD	Minimum	Maximum
Spectral clustering	0.99	0.03	0.80	1.00
Hierarchical clustering	0.98	0.09	0.32	1.00
HDBSCAN	0.87	0.09	0.61	1.00
K-Prototypes clustering	0.67	0.19	0.42	1.00
Consensus clustering	0.92	0.10	0.62	1.00

Adjusted Rand Index (ARI) values are reported for 50 bootstrap resamples for each clustering method. Mean, standard deviation (SD), minimum, and maximum are shown.

Spectral clustering and hierarchical clustering demonstrated very high stability (mean ARI > 0.98). HDBSCAN showed good stability (mean ARI = 0.88), while K-Prototypes clustering showed moderate stability with higher variability (mean ARI = 0.67).

Following the development of four clustering algorithms, consensus clustering was performed to combine their complementary information and strengths.

### 3.6 Consensus clustering

**Figure 4:**
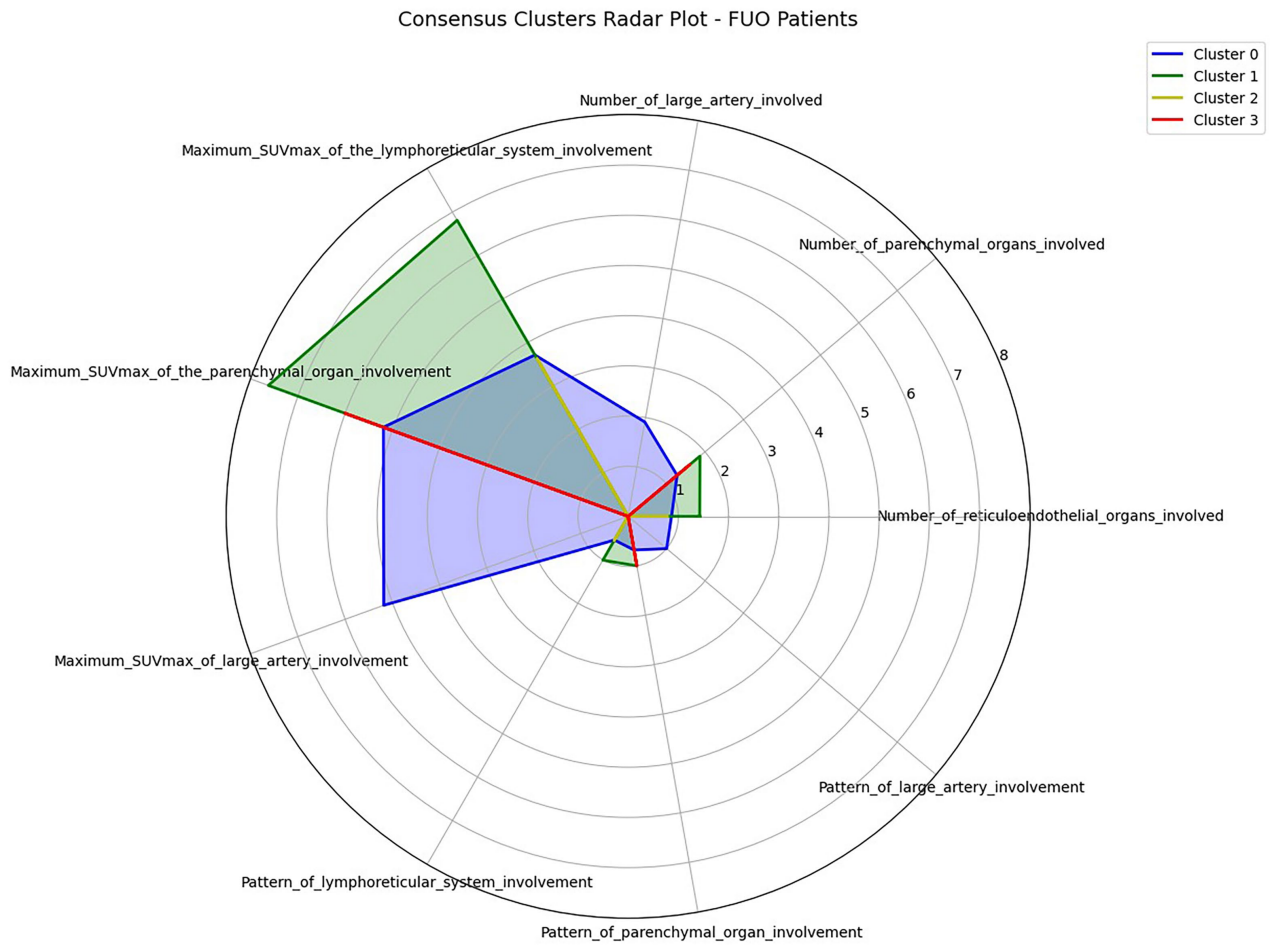
Radar plot showing key PET/CT features across the four clusters. Each cluster displays a distinct imaging profile.

The PET/CT characteristics of the groups obtained through consensus clustering are summarized in Figure 4 and Table 2.

**Table 2:** PET/CT features of FUO patients across consensus-defined clusters.

Clusters	Number of reticuloendothelial organs involved	Number of parenchymal organs involved	Number of large artery involved	Maximum SUVmax of the lymphoreticular system involvement	Maximum SUVmax of the parenchymal organ involvement	Maximum SUVmax of large artery involvement	Pattern of lymphoreticular system involvement	Pattern of parenchymal organ involvement	Pattern of large artery involvement
0	0.86	1.27	1.91	3.71	5.19	5.18	No	Focal	Focal
1	1.42	1.87	0.00	6.81	7.63	0.00	Focal	Focal	No
2	0.78	0.00	0.00	3.62	0.00	0.00	No	No	No
3	0.00	1.60	0.00	0.00	6.00	0.00	No	Focal	No

Cluster 0 represents unclassified patients or the general population.

Consensus clustering of PET/CT features in FUO patients identified four distinct clusters reflecting heterogeneous patterns of organ involvement and metabolic activity. Cluster 0 consisted of 44 patients and included cases that could not be clearly assigned to a specific group. This cluster showed moderate involvement of reticuloendothelial organs, parenchymal organs, and large arteries, with focal uptake observed in parenchymal organs and large arteries. Cluster 1 was characterized by prominent reticuloendothelial and parenchymal organ involvement with focal uptake in both compartments, whereas large arteries were largely spared. Cluster 2 showed mild involvement of reticuloendothelial organs with no focal or diffuse uptake, while parenchymal organs and large arteries remained unaffected. Cluster 3 demonstrated selective parenchymal organ involvement with focal uptake, whereas reticuloendothelial organs and large arteries were largely uninvolved. These clusters collectively illustrate the spectrum of PET/CT phenotypes in FUO patients, ranging from mild single-organ involvement to extensive multi-organ uptake.

**Figure 5:**
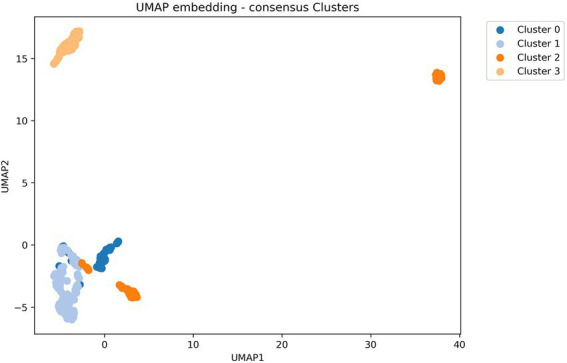
Two-dimensional UMAP (Uniform Manifold Approximation and Projection) visualization of 289 FUO patients based on PET/CT-derived imaging features. Each point represents an individual patient, and colors indicate the consensus cluster assignment obtained from the clustering analysis. Patients positioned closer together in the plot have more similar PET/CT feature profiles, whereas patients located farther apart are more dissimilar. The clear spatial separation between clusters demonstrates that the identified consensus clusters represent distinct PET/CT phenotypic patterns among FUO patients. Axes (UMAP1 and UMAP2) represent the low-dimensional embedding coordinates generated by the UMAP algorithm and do not correspond to direct clinical variables.

Clusters obtained from consensus clustering are visualized in a UMAP plot generated from PET/CT-derived features (Figure 5), demonstrating their separation.

The UMAP embedding demonstrates a clear separation for Cluster 3, which appears as a well-defined and internally homogeneous group. In contrast, Clusters 0, 1, and 2 are located in close proximity, showing substantial overlap and less distinct boundaries, suggesting shared features and partial intermixing among these groups.

The consensus clustering solution showed high stability, with a mean ARI of 0.93 (SD 0.10), ranging from 0.63 to 1.00 (Table 1, Figure 3). Overall, clustering stability was high across all methods except for K-prototypes, which demonstrated moderate variability. Importantly, the consensus clustering approach, which integrates all methods, also showed high stability and was used as the primary clustering framework in our analysis.

The consensus clustering approach identified four distinct patient clusters. To determine whether PET/CT-derived features differed among these clusters, statistical comparisons were performed for both continuous/ordinal and categorical variables.

### 3.7 Statistical comparison of PET/CT-derived features across consensus clusters

Two complementary approaches were used to compare PET/CT-derived features among the consensus clustering groups. First, statistical hypothesis testing was performed using the Kruskal–Wallis test for continuous and ordinal variables and the chi-square test of independence for categorical variables. Second, the magnitude of differences between clusters was evaluated using SMD.

#### 3.7.1 Statistical comparison of PET/CT-derived features across consensus clusters using hypothesis testing

All continuous and ordinal PET/CT features demonstrated statistically significant differences across clusters according to the Kruskal–Wallis test (all *p* < 0.05). Post-hoc analysis using Dunn’s test with Bonferroni correction identified significant pairwise differences between multiple cluster combinations for each variable. Detailed results are presented in Table 3.

**Table 3:** Comparison of continuous PET/CT-derived variables among consensus clusters using the Kruskal–Wallis test.

Feature	Kruskal-wallis *χ^2^*	*p*-value	Post-hoc (significant pairwise)
Number of large arteries involved	285.7	1.24 × 10^−61^	0 vs. 1, 0 vs. 2, 0 vs. 3
Maximum SUVmax of large artery involvement	285.41	1.43 × 10^−61^	0 vs. 1, 0 vs. 2, 0 vs. 3
Number of parenchymal organs involved	158.03	4.89 × 10^−34^	0 vs. 2, 1 vs. 2, 2 vs. 3
Maximum SUVmax of parenchymal organ involvement	145.65	2.29 × 10^−31^	0 vs. 2, 1 vs. 2, 2 vs. 3
Number of reticuloendothelial organs involved	135.48	3.56 × 10^−29^	0 vs. 1, 0 vs. 3, 1 vs. 2, 1 vs. 3, 2 vs. 3
Maximum SUVmax of lymphoreticular system involvement	129.63	6.51 × 10^−28^	0 vs. 1, 0 vs. 3, 1 vs. 2, 1 vs. 3, 2 vs. 3

Pairwise comparisons were performed using Dunn’s post-hoc test with Bonferroni correction. Only statistically significant pairwise comparisons (*p* < 0.05) are reported.

Similarly, categorical PET/CT features, including organ involvement and involvement patterns, also differed significantly between clusters based on the chi-square test of independence (all *p* < 0.05). Pairwise comparisons using chi-square tests with Bonferroni correction revealed significant differences between several cluster pairs. These results are summarized in Table 4.

**Table 4:** Comparison of categorical PET/CT variables, including organ involvement and involvement patterns, among consensus clusters using the chi-square test of independence.

Feature	Chi-square *χ^2^*	*p*-value	Pairwise comparisons (significant)
Lymphoreticular system involvement	166.94	5.81 × 10^−36^	1 vs. 2, 1 vs. 0, 1 vs. 3, 2 vs. 3, 0 vs. 3
Pattern of lymphoreticular system involvement	172.18	1.55 × 10^−34^	1 vs. 2, 1 vs. 0, 1 vs. 3, 2 vs. 3, 0 vs. 3
Parenchymal organ involvement	241.68	4.12 × 10^−52^	1 vs. 2, 1 vs. 0, 2 vs. 0, 2 vs. 3, 0 vs. 3
Pattern of parenchymal organ involvement	241.68	4.12 × 10^−52^	1 vs. 2, 1 vs. 0, 2 vs. 0, 2 vs. 3, 0 vs. 3
Large artery involvement	289.0	2.39 × 10^−62^	1 vs. 0, 2 vs. 0, 0 vs. 3
Pattern of large artery involvement	289.0	2.39 × 10^−62^	1 vs. 0, 2 vs. 0, 0 vs. 3

Significant pairwise comparisons between clusters were identified using chi-square tests with Bonferroni correction. Only statistically significant comparisons (*p* < 0.05) are shown.

Overall, these findings indicate that the consensus clusters represent distinct patient groups with significantly different PET/CT involvement characteristics across all evaluated variables.

#### 3.7.2 Effect size analysis using standardized mean differences

The magnitude of differences between clusters was further evaluated using SMD, which demonstrated large effect sizes for several PET/CT variables (Table 5).

**Table 5:** Standardized mean differences (SMD) for pairwise comparisons of PET/CT continuous and ordinal features across the four consensus clusters.

Feature	Cluster comparison	SMD
Number of reticuloendothelial organs involved	1 vs. 2	0.86
	1 vs. 0	0.70
	1 vs. 3	3.27
	2 vs. 0	−0.10
	2 vs. 3	1.27
	0 vs. 3	1.28
Maximum SUVmax of the lymphoreticular system involvement	1 vs. 2	0.58
	1 vs. 0	0.64
	1 vs. 3	1.79
	2 vs. 0	−0.02
	2 vs. 3	0.91
	0 vs. 3	1.23
Number of parenchymal organs involved	1 vs. 2	3.00
	1 vs. 0	0.57
	1 vs. 3	0.31
	2 vs. 0	−1.51
	2 vs. 3	−2.86
	0 vs. 3	−0.33
Maximum SUVmax of parenchymal organ involvement	1 vs. 2	1.52
	1 vs. 0	0.38
	1 vs. 3	0.31
	2 vs. 0	−1.26
	2 vs. 3	−3.39
	0 vs. 3	−0.18
Number of large arteries involved	1 vs. 2	NA
	1 vs. 0	−3.37
	1 vs. 3	NA
	2 vs. 0	−3.37
	2 vs. 3	NA
	0 vs. 3	3.37
Maximum SUVmax of large artery involvement	1 vs. 2	NA
	1 vs. 0	−3.24
	1 vs. 3	NA
	2 vs. 0	−3.24
	2 vs. 3	NA

Each row represents a cluster-to-cluster comparison for a given feature. SMD values indicate the magnitude and direction of difference between clusters. SMD: standardized mean difference. Values of 0.2, 0.5, and 0.8 are commonly interpreted as small, medium, and large effect sizes, respectively. NA values indicate comparisons where the standard deviation was zero in one or both clusters, preventing SMD calculation (NA: Not applicable).

After examining the differences in variables across consensus clusters, the distribution of final diagnoses among the clusters was evaluated to assess the relationship between cluster membership and underlying etiology.

### 3.8 Distribution of final diagnoses across consensus clusters

**Figure 6:**
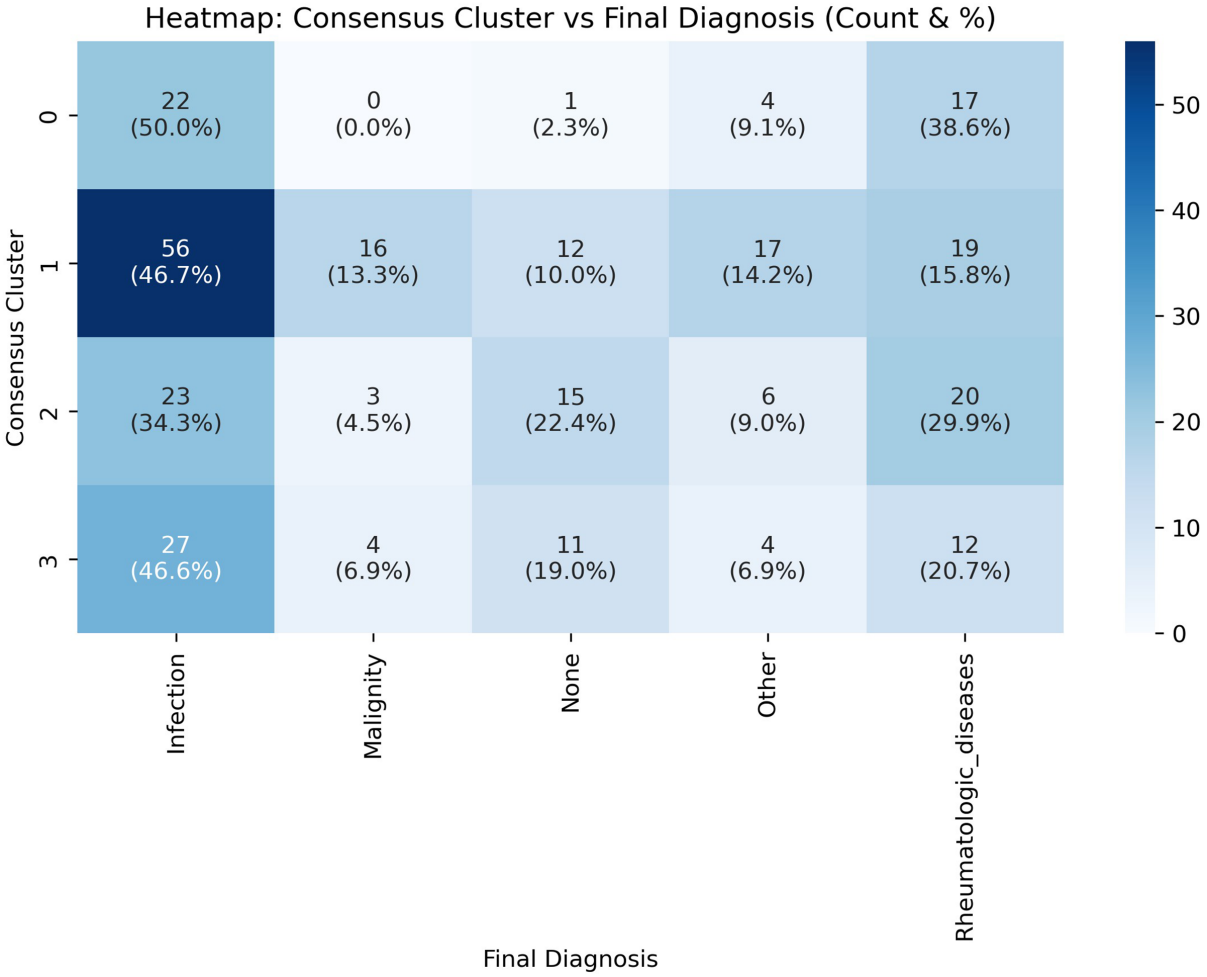
Heatmap showing the distribution of final diagnoses across clusters. Each cell represents the number of patients within a cluster for a given diagnosis group.

The distribution of final diagnoses according to the groups obtained by consensus clustering is summarized in Table 6 and Figure 6. Cluster 0 was characterized by involvement of the reticuloendothelial, parenchymal, and large arterial systems, and was the only cluster demonstrating arterial involvement. Cluster 1 showed combined reticuloendothelial and parenchymal involvement, Cluster 2 was predominantly characterized by reticuloendothelial involvement, and Cluster 3 primarily exhibited parenchymal involvement. Across all clusters, infections accounted for approximately half of the cases (34.3–50%), while rheumatologic diseases represented around 15.8–29.9% in all clusters except Cluster 2, where they were more prominent, reaching 38.6%. Malignancies and other conditions were observed at lower proportions across all groups.

**Table 6:** Distribution of final diagnoses across consensus-defined clusters of FUO patients.

Clusters	Infection	Malignancy	None	Other	Rheumatologic_diseases
0	56 (46.7)	16 (13.3)	12 (10.0)	17 (14.2)	19 (15.8)
1	27 (46.6)	4 (6.9)	11 (19.0)	4 (6.9)	12 (20.7)
2	22 (50.0)	0 (0.0)	1 (2.3)	4 (9.1)	17 (38.6)
3	23 (34.3)	3 (4.5)	15 (22.4)	6 (9.0)	20 (29.9)

Cluster 0 represents unclassified patients or the general population.

The comparison between the consensus clustering assignments and the final clinical diagnosis showed a low to moderate agreement. ARI was 0.006, indicating minimal overall overlap between cluster membership and diagnosis. NMI was 0.047, consistent with weak correspondence. Statistical association was significant (Chi-square = 32.46, *p* = 0.0012, degrees of freedom = 12), but the effect size measured by Cramér’s V was low (*V* = 0.19), suggesting that while the association exists, clusters do not strongly align with the final diagnoses.

Representative cases from each consensus-defined cluster are presented in Table 7 to illustrate the corresponding clinical and PET/CT patterns. The selected cases further highlight the heterogeneity of imaging patterns within each diagnostic category.

**Table 7:** Representative cases demonstrating the clinical and PET/CT characteristics of consensus-defined clusters.

Sample cases	Final diagnosis	Cluster	Parenchymal involvement	Reticuloendothelial involvement	Large artery involvement	PET/CT findings
1	Non-Hodgkin lymphoma	1	+	+	−	Cervical, axillary, mediastinal, and abdominal lymphadenopathy; splenic and bone marrow involvement; uptake in the left femoral greater trochanter; pulmonary nodules
2	Tuberculous lymphadenopathy	1	+	+	−	Cervical, mediastinal, and intra-abdominal lymphadenopathy; focal uptake in the lower pole of the left kidney; nodular pulmonary involvement in bilateral apicoposterior regions
3	Takayasu arteritis	0	+	+	+	Diffuse involvement of the aorta and its major branches; cervical and mediastinal lymphadenopathy; left salivary gland involvement
4	Sarcoidosis	1	+	+	−	Mediastinal lymphadenopathy; bilateral parotid gland involvement; nodular lesions in the right lung apex and posterobasal segment of the lower lobe
5	Subcutaneous abscess	3	+	−	−	Subcutaneous abscess at the sacral region
6	T-cell lymphoma	2	−	+	−	Mediastinal and intra-abdominal lymphadenopathy; splenic and bone marrow involvement
7	Still	2	−	+	−	Mediastinal lymphadenopathy, splenic and bone marrow involvement

## 4 Discussion

Fever of unknown origin remains a major diagnostic challenge because of its heterogeneous etiologies and non-specific clinical presentation (1). Although PET/CT has become an important imaging modality in the evaluation of FUO, most previous studies have assessed its usefulness primarily in terms of its ability to localize abnormal sites. The actual diagnosis of disease at these PET-positive sites is confirmed using targeted laboratory tests, imaging, or biopsy; thus, PET/CT alone does not directly establish the underlying diagnosis (2–10). However, only a limited number of studies have attempted to directly predict the underlying etiology using PET/CT imaging features such as SUVmax and the anatomical distribution of uptake (11–14), representing the approaches most closely related to our methodology. To our knowledge, none of these studies has applied machine learning–based clustering to group FUO patients solely according to PET/CT features and subsequently relate these groups to final diagnoses in order to identify imaging phenotypes that may predict underlying etiologies. Therefore, this approach represents a novel contribution to the FUO literature.

In this study, we investigated whether FUO patients could be classified solely based on PET/CT-derived features. Using mixed numerical and categorical variables describing organ involvement, uptake distribution, and SUVmax values, we applied multiple unsupervised clustering algorithms to identify potential imaging-based phenotypes among 289 FUO patients. Despite the heterogeneous structure of the dataset, clustering analyses revealed reproducible patterns with significant differences in PET/CT characteristics across groups.

Bootstrap analysis demonstrated that spectral and hierarchical clustering showed very high stability, HDBSCAN showed good stability, and K-prototypes showed moderate stability. The preservation of clustering structures across bootstrap resamples indicates that the models were robust and supports the reliability of the identified clustering patterns. Despite appropriate preprocessing (standardization and encoding), K-prototypes exhibited only moderate stability. This is likely due to its sensitivity to initial cluster assignments, which can be amplified in datasets with heterogeneous mixed-type variables, leading to variability in cluster membership across bootstrap resamples (15). In contrast, Gower distance-based methods (spectral clustering, hierarchical clustering, HDBSCAN) are grounded in using Gower’s dissimilarity—a metric widely adopted for clustering mixed continuous and categorical data because it simultaneously accommodates both data types in a single similarity measure—and have been shown to provide consistent performance in mixed-type clustering contexts (18).

In our study, consensus clustering was applied to integrate the results of the four clustering algorithms, producing a robust final clustering structure. By combining the complementary strengths of each method and mitigating the limitations of individual algorithms, this approach enhanced stability and overall robustness compared with any single algorithm, consistent with previous findings that consensus methods improve consistency and reliability by aggregating multiple clustering results (19). This robustness was further supported by bootstrap analysis, which demonstrated high stability of the consensus clustering solution.

Within this consensus structure, Cluster 0 involved the reticuloendothelial, parenchymal, and large arterial systems, and was the only cluster demonstrating arterial involvement. Cluster 1 involved both reticuloendothelial and parenchymal organs, Cluster 2 showed predominantly reticuloendothelial involvement, and Cluster 3 displayed parenchymal organ uptake. In the UMAP projection used for visualization, Cluster 3 appeared relatively more compact and separated compared with the other clusters. In contrast, Clusters 0, 1, and 2 were closely positioned with substantial overlap, indicating shared PET/CT features and overlapping patterns of organ involvement. It should be noted that UMAP provides a two-dimensional visualization of high-dimensional relationships, and therefore the apparent distances or separations between clusters in the projection may not fully represent the relationships in the original feature space, as dimensionality-reduction techniques such as UMAP may distort global structures in the embedding (20).

Infections were the most common etiologic category across all clusters, followed by rheumatologic diseases. From a pathophysiological perspective, infections can involve virtually any parenchymal organ and may be accompanied by local or systemic reticuloendothelial system (RES) activation. Accordingly, the patterns observed in Clusters 0, 1, and 3, characterized by combined parenchymal and/or RES involvement, can be readily explained by infectious processes. In addition, certain infectious diseases may preferentially involve the reticuloendothelial system, which may contribute to RES-predominant uptake patterns observed in some FUO patients (16, 21, 22).

However, the presence of vascular involvement in Cluster 0 warrants cautious interpretation. Although large-vessel FDG uptake is classically associated with primary vasculitic conditions, it is not disease-specific. A spectrum of infectious processes may also result in vascular metabolic activity on PET/CT, potentially mediated by mechanisms such as endothelial activation driven by systemic inflammatory cytokines, direct microbial endothelial invasion, or entities such as infectious aortitis and other endovascular infections, which may exhibit similar vascular uptake patterns (23–28). Accordingly, vascular FDG uptake should be interpreted within a broad differential diagnostic framework rather than being attributed exclusively to primary vasculitis.

Rheumatologic diseases also have the potential to involve parenchymal organs, the reticuloendothelial system, and large vessels, depending on the underlying disease spectrum. Large-vessel vasculitides are typically associated with vascular FDG uptake on PET/CT, whereas small and medium-vessel inflammatory diseases may present with predominant parenchymal involvement, often accompanied by varying degrees of reticuloendothelial activation, while some rheumatologic conditions such as Adult-onset Still’s disease may be characterized by predominant reticuloendothelial involvement with limited parenchymal and vascular uptake (17, 29–32). In line with this heterogeneous organ involvement, rheumatologic diseases were distributed across all clusters and consistently represented the second most common etiologic category, underscoring their capacity to produce diverse imaging patterns.

Overall, the predominance of infections across clusters, as well as the ranking of rheumatologic diseases as the second most frequent etiology, may reflect the underlying distribution of cases in our cohort (infections: 128/289, 44.3%; rheumatologic diseases: 68/289, 23.5%; malignancies: 23/289, 8%; other or unclassified causes: 31/289, 10.7%). Literature on PET/CT-based comparison of etiologic causes according to uptake patterns and SUVmax is relatively limited (11–14). Several studies have suggested that PET/CT features may provide supportive information for FUO etiologies, with higher SUVmax and imaging patterns such as asymmetric lymphadenopathy, increased bone marrow or splenic uptake, and focal hepatic involvement more frequently reported in malignancy, whereas lower SUVmax values have been described in infections and rheumatologic diseases (11–14), reflecting a general tendency of PET/CT to demonstrate different uptake patterns across disease categories. In these studies, malignant diseases are generally reported to show more distinct imaging patterns compared with infectious and rheumatologic conditions.

In contrast to these studies, which primarily evaluate etiologic differences based on PET/CT uptake patterns and SUVmax, our study adopted a different approach in which patients were stratified into clusters according to PET/CT imaging patterns, and the distribution of etiologic categories across these clusters was subsequently assessed. No clear differences in etiologic composition were observed between clusters. This may be partly explained by the limited number of malignancy cases and the predominance of infectious and rheumatologic diseases in our cohort, which may have influenced the ability of SUVmax and uptake pattern–based clustering to discriminate between etiologic categories.

An important methodological consideration is that the dataset itself did not demonstrate a strong intrinsic clustering tendency, as reflected by the relatively low Hopkins statistic (*H* = 0.37). In addition, the Gower distance heatmap revealed a heterogeneous structure without sharply separated patient groups. These findings suggest that the underlying PET/CT patterns in FUO may exist along a continuum rather than forming clearly distinct natural clusters. This may partly explain the substantial overlap observed between consensus clusters and final diagnostic categories, as different etiologies can share similar patterns of metabolic activity and organ involvement. Therefore, the identified clusters should be interpreted primarily as broad imaging phenotypes rather than strictly separated disease-specific entities.

Accordingly, comparison of consensus clusters with final clinical diagnoses showed limited concordance. Although the association between clusters and final diagnoses reached statistical significance (Chi-square = 32.46, *p* = 0.0012), the effect size was weak (Cramér’s V = 0.19), and agreement remained minimal according to ARI (0.006) and NMI (0.047). These findings suggest that PET/CT-based clusters primarily capture imaging phenotypes rather than disease-specific diagnostic categories, as different etiologies—particularly rheumatologic conditions and atypical infections—may present with overlapping uptake patterns. In addition, the predominance of infections in our cohort may have further influenced this result. Therefore, clustering should be viewed as a tool for identifying imaging patterns that complement, rather than replace, clinical diagnosis.

Before clustering, we hypothesized that specific PET/CT uptake patterns might reflect underlying etiologic mechanisms—for example, parenchymal involvement with focal or diffuse reticuloendothelial activation in infections and small-vessel vasculitides; vascular uptake in large-vessel vasculitides; and focal or multifocal parenchymal organ involvement with associated lymphadenopathy in solid organ malignancies, whereas lymphomas were expected to show predominant reticuloendothelial involvement. However, the relatively high proportion of infectious and rheumatologic diseases in our cohort—both of which can involve the reticuloendothelial system and parenchymal organs and often present with multisystem involvement—may have limited the ability of this conceptual framework to clearly separate diagnostic groups.

Our study has several limitations. First, the cohort was predominantly composed of infectious cases, which may have limited the representation of rarer etiologies and contributed to the modest alignment between clusters and final diagnoses. Second, the retrospective and single-center design may introduce selection and information biases and limit the generalizability of our findings. Third, PET/CT parameters such as SUVmax can be influenced by scanner type, acquisition timing, and administered dose. In addition, organ involvement patterns were visually assessed by a single experienced nuclear medicine physician, which may introduce observer-related variability and limit assessment of interobserver reproducibility. Fourth, methodological aspects of cluster analysis—including algorithm selection, parameter tuning, and the heterogeneity of mixed-type data—may affect cluster stability, while dimensionality reduction for visualization (e.g., UMAP) may exaggerate or obscure certain relationships. In addition, FUO and IUO patients were analyzed within the same clustering framework. Previous studies have suggested that FDG-PET/CT may provide higher diagnostic utility in IUO patients compared with FUO patients, potentially because IUO cohorts may contain a higher proportion of rheumatologic and large-vessel inflammatory diseases, in which PET/CT is particularly useful for detecting vascular inflammation and characteristic uptake patterns (33, 34). Although no significant difference was observed between FUO and IUO patients regarding etiologic distribution in our cohort, differences in PET/CT involvement patterns were identified, with FUO patients demonstrating more frequent large artery involvement and less frequent parenchymal involvement compared with IUO patients. Because clustering algorithms group patients according to similarity in imaging features, these differences may have increased imaging heterogeneity and influenced cluster separability. Consequently, inclusion of both FUO and IUO patients may have contributed to the limited concordance observed between clusters and final diagnostic categories. Fifth, PET/CT images were evaluated by a single nuclear medicine physician, and interobserver agreement was not assessed. Sixth, the absence of an independent external validation cohort is a limitation of this study. Although bootstrap-based internal validation demonstrated high clustering stability, the findings were derived from a single retrospective cohort and therefore require confirmation in independent multicenter populations before broader generalization. Finally, small numbers of cases in some etiologic subgroups, particularly malignancies and rare rheumatologic diseases, may have limited statistical power for cluster characterization.

In conclusion, consensus clustering of organ-specific PET/CT uptake patterns in FUO patients identified reproducible imaging-based patterns characterized by different distributions of parenchymal, reticuloendothelial, and vascular involvement. However, the agreement between imaging-based clusters and final clinical diagnoses was limited, indicating that these clusters primarily reflect overlapping metabolic imaging patterns rather than disease-specific etiologic categories. These findings support the complementary role of PET/CT in FUO evaluation and suggest that clustering approaches may help characterize heterogeneous imaging presentations while requiring careful clinical interpretation.

## Data Availability

The data analyzed in this study is subject to the following licenses/restrictions: patient data is kept confidential. Requests to access these datasets should be directed to Istanbul Medipol University.

## Supplementary material

SUPPLEMENTARY FIGURE S1 Heatmap of the Gower distance matrix showing pairwise similarity among 289 patients with fever of unknown origin (FUO) based on PET/CT-derived imaging features. Each row and column represents an individual patient. The color intensity corresponds to the degree of dissimilarity between patients: darker colors indicate lower Gower distances (greater similarity in PET/CT patterns), whereas lighter colors indicate higher distances (greater differences in imaging characteristics). The diagonal line represents self-comparisons, where the distance is zero. Visual clustering patterns suggest the presence of subgroups of patients with similar PET/CT involvement profiles.

SUPPLEMENTARY FIGURE S2 Hierarchical clustering dendrogram of 289 FUO patients generated using Gower distance and agglomerative hierarchical clustering of PET/CT-derived variables. Each terminal branch represents an individual patient, while the vertical height of branch junctions reflects the degree of dissimilarity between patients or patient groups. Patients that merge at lower heights are more similar in terms of PET/CT findings, whereas those joining at higher levels are more distinct. Colored branches indicate the major clusters identified by the clustering algorithm, representing groups of patients with shared PET/CT involvement patterns.

SUPPLEMENTARY FIGURE S3 UMAP plot showing the distribution of patients based on hierarchical clustering. Each point represents a patient, colored by cluster. The plot illustrates cluster separation and internal structure.

SUPPLEMENTARY FIGURE S4 UMAP plot showing the distribution of patients based on HDBSCAN clustering. Each point represents a patient, colored by cluster. Grey points indicate patients not assigned to any cluster (noise).

SUPPLEMENTARY FIGURE S5 UMAP plot showing the distribution of patients based on spectral clustering. Each point represents a patient, colored by cluster.

SUPPLEMENTARY FIGURE S6 UMAP plot showing the distribution of patients based on K-prototypes clustering. Each point represents a patient, colored by cluster. The plot illustrates cluster separation.
