# Supplementary figures and images for: Unsupervised clustering of PET/CT features in fever of unknown origin (FUO) and inflammation of unknown origin (IUO)
Source: Front Med (Lausanne). 2026 May 29;13:1830800. doi: 10.3389/fmed.2026.1830800 (PMC13259882; doi:10.3389/fmed.2026.1830800)

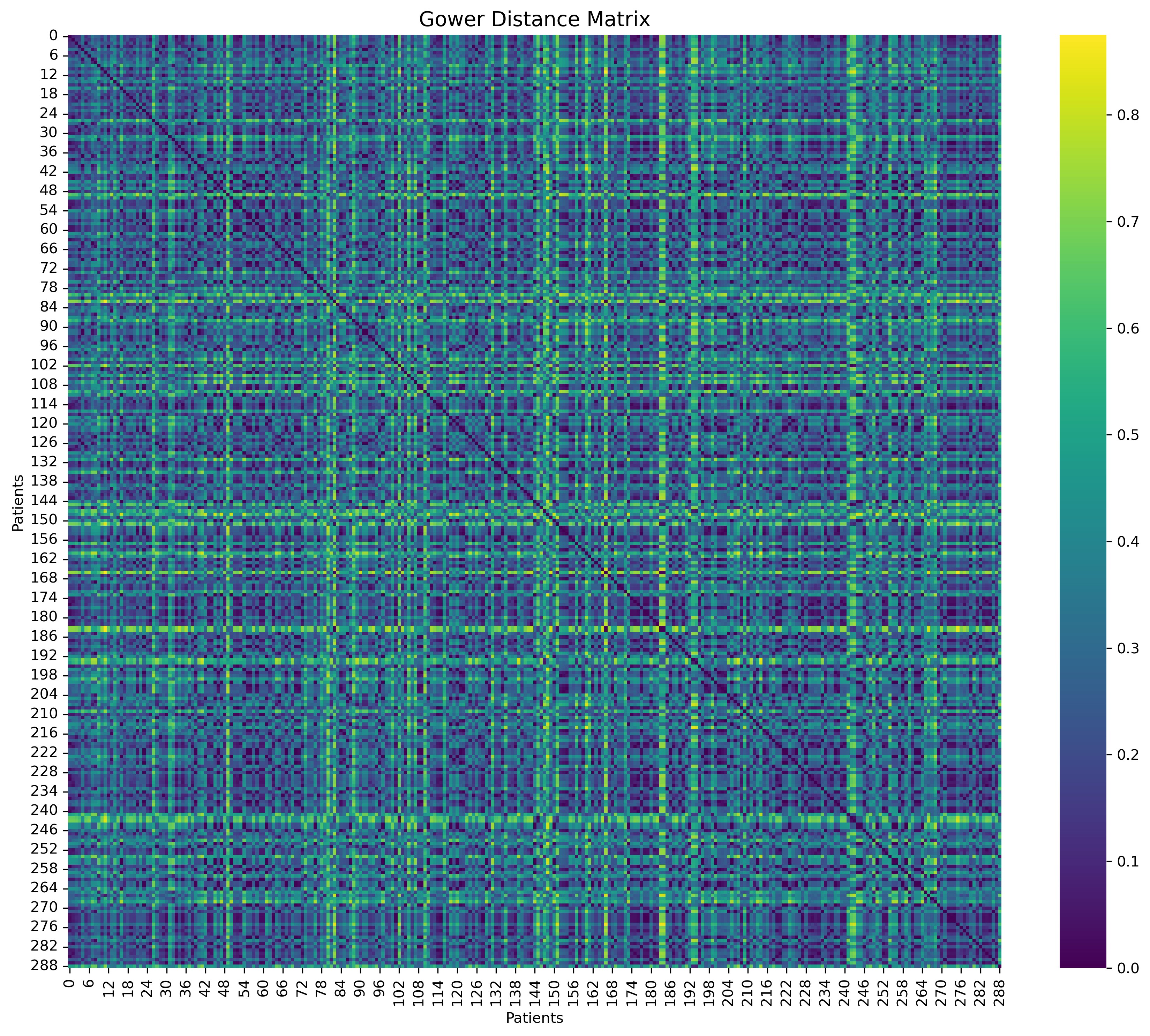

Supplement: SUPPLEMENTARY FIGURE S1 — Heatmap of the Gower distance matrix showing pairwise similarity among 289 patients with fever of unknown origin (FUO) based on PET/CT-derived imaging features. Each row and column represents an individual patient. The color intensity corresponds to the degree of dissimilarity between patients: darker colors indicate lower Gower distances (greater similarity in PET/CT patterns), whereas lighter colors indicate higher distances (greater differences in imaging characteristics). The diagonal line represents self-comparisons, where the distance is zero. Visual clustering patterns suggest the presence of subgroups of patients with similar PET/CT involvement profiles. [file Image_1.jpeg]

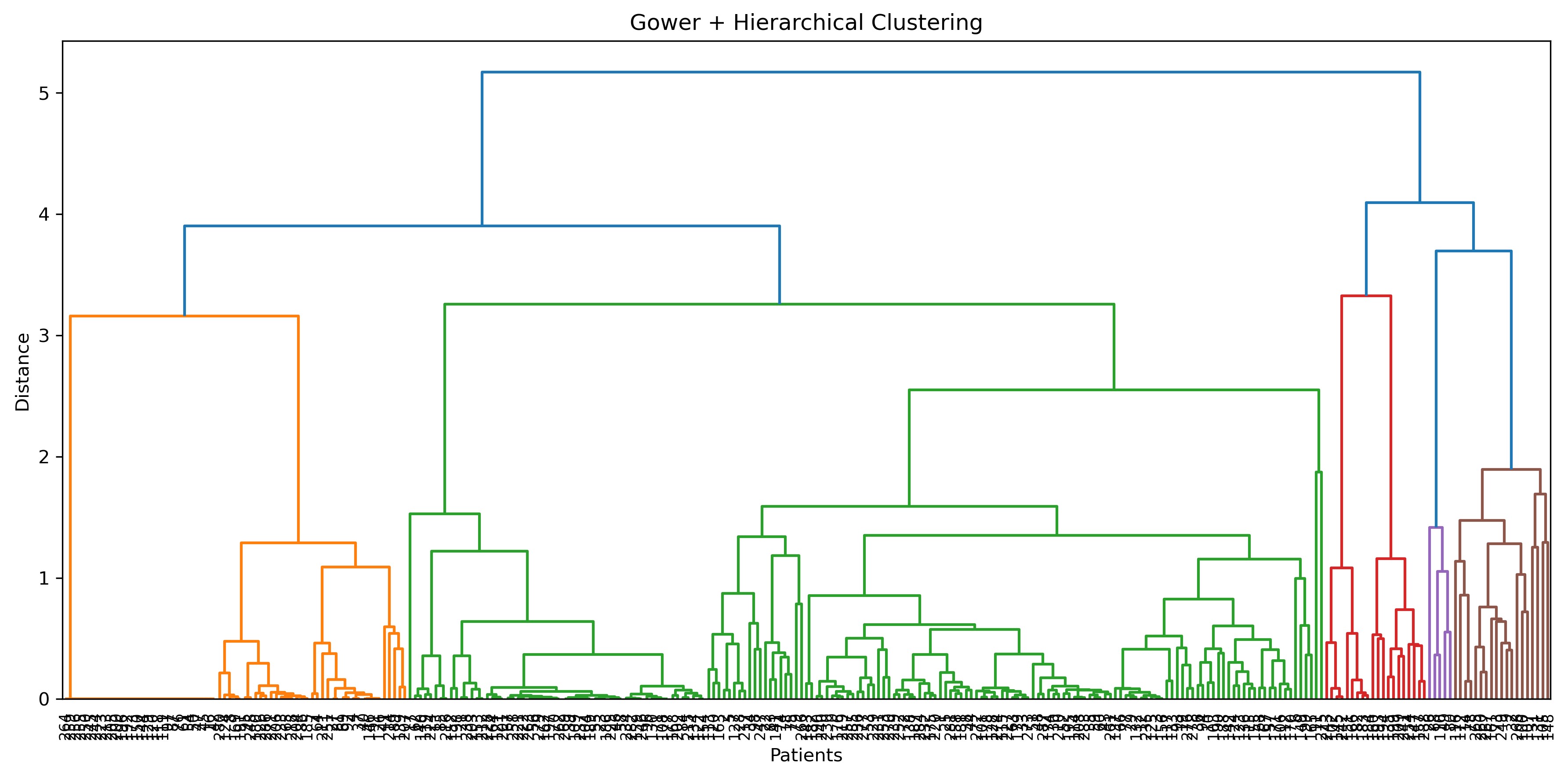

Supplement: SUPPLEMENTARY FIGURE S2 — Hierarchical clustering dendrogram of 289 FUO patients generated using Gower distance and agglomerative hierarchical clustering of PET/CT-derived variables. Each terminal branch represents an individual patient, while the vertical height of branch junctions reflects the degree of dissimilarity between patients or patient groups. Patients that merge at lower heights are more similar in terms of PET/CT findings, whereas those joining at higher levels are more distinct. Colored branches indicate the major clusters identified by the clustering algorithm, representing groups of patients with shared PET/CT involvement patterns. [file Image_2.jpeg]

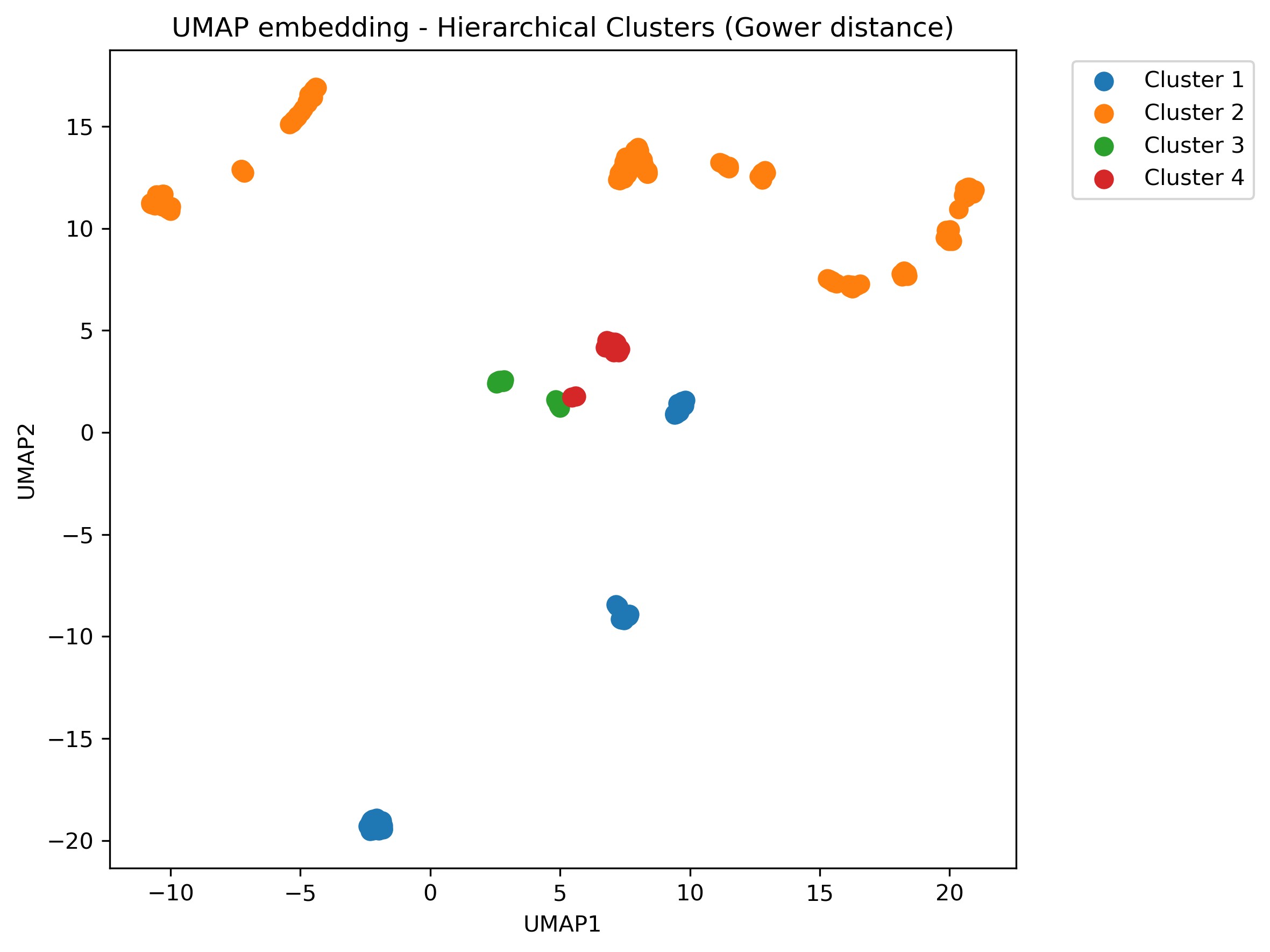

Supplement: SUPPLEMENTARY FIGURE S3 — UMAP plot showing the distribution of patients based on hierarchical clustering. Each point represents a patient, colored by cluster. The plot illustrates cluster separation and internal structure. [file Image_3.jpeg]

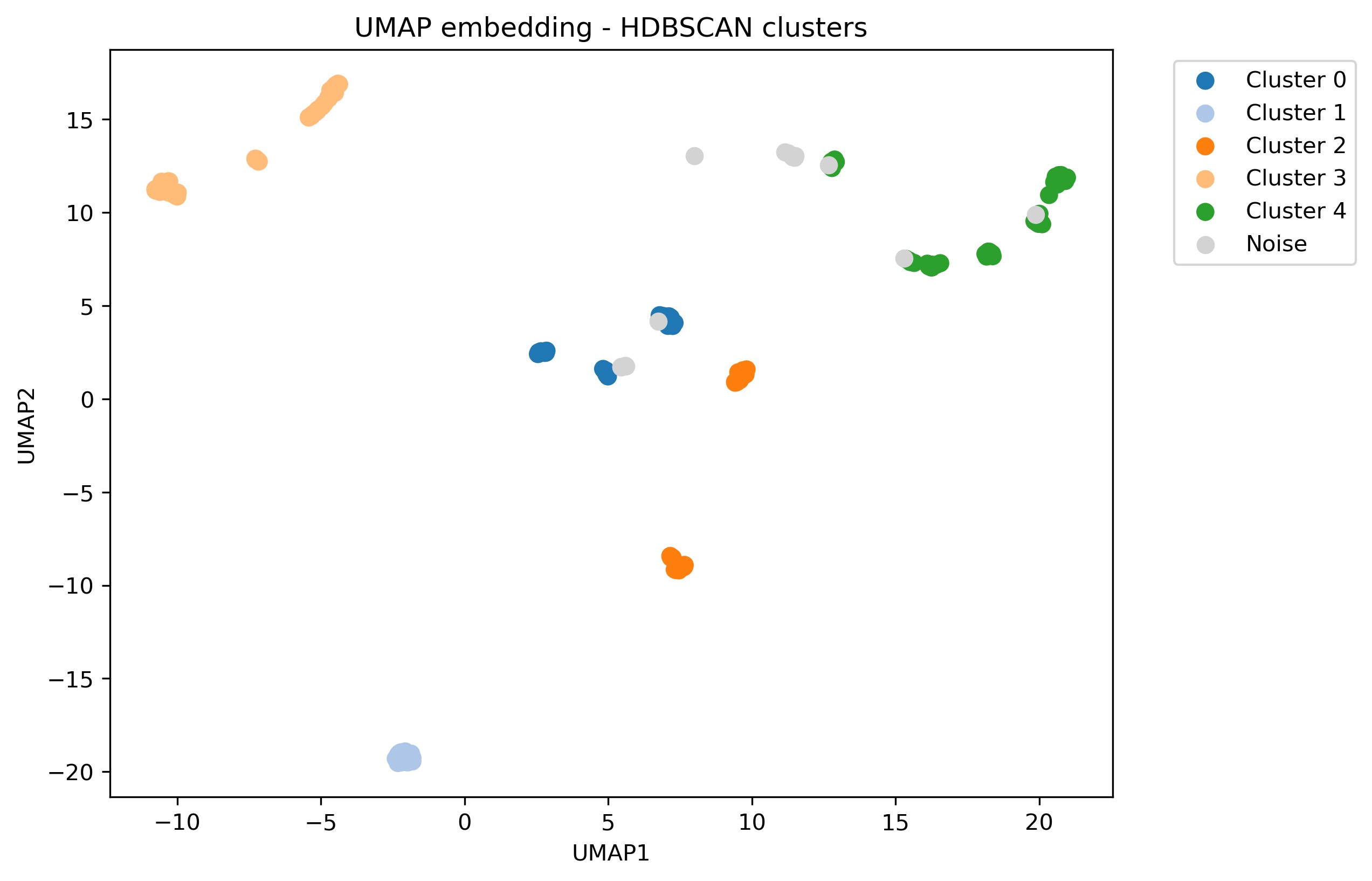

Supplement: SUPPLEMENTARY FIGURE S4 — UMAP plot showing the distribution of patients based on HDBSCAN clustering. Each point represents a patient, colored by cluster. Grey points indicate patients not assigned to any cluster (noise). [file Image_4.jpeg]

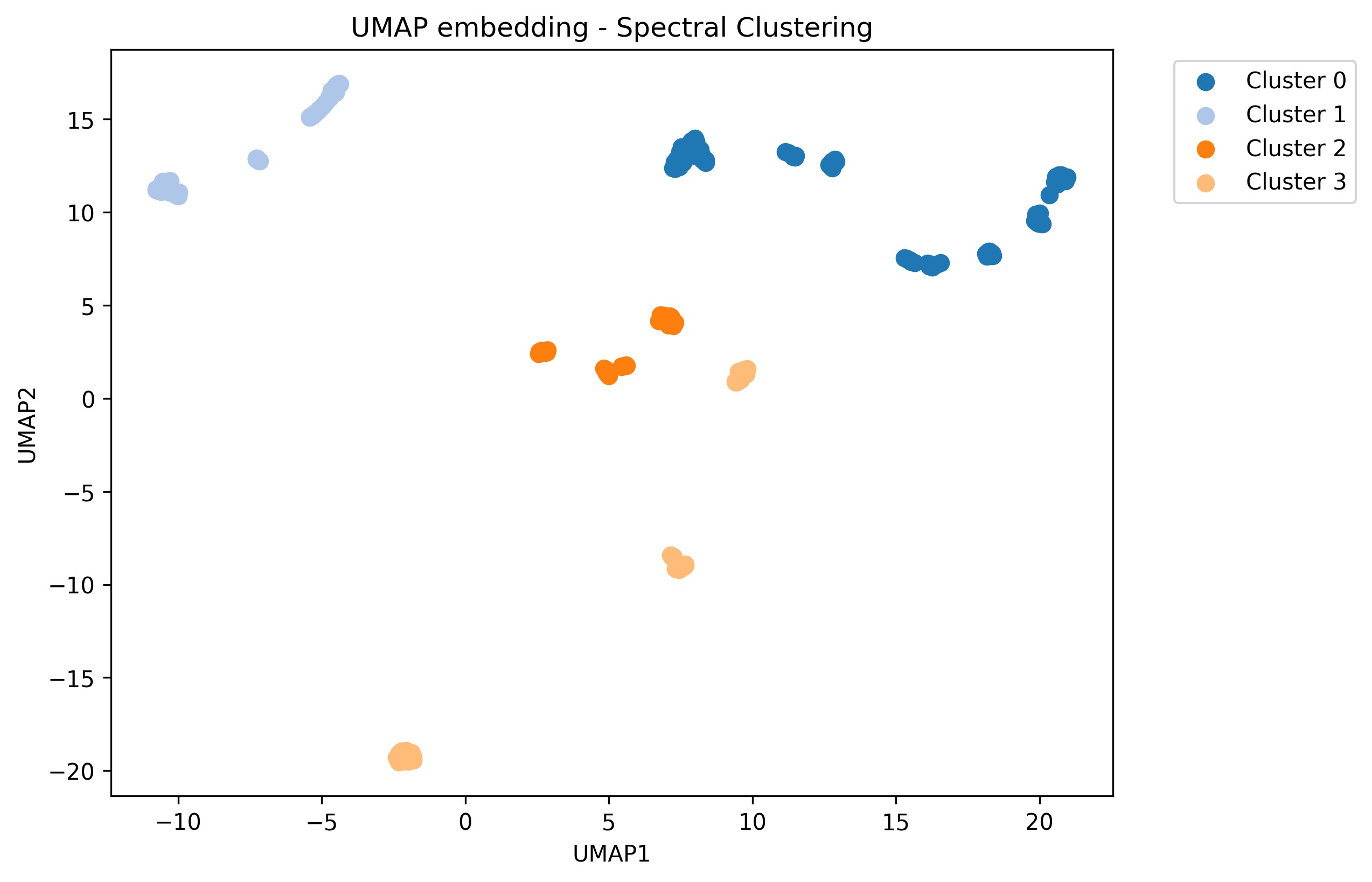

Supplement: SUPPLEMENTARY FIGURE S5 — UMAP plot showing the distribution of patients based on spectral clustering. Each point represents a patient, colored by cluster. [file Image_5.jpeg]

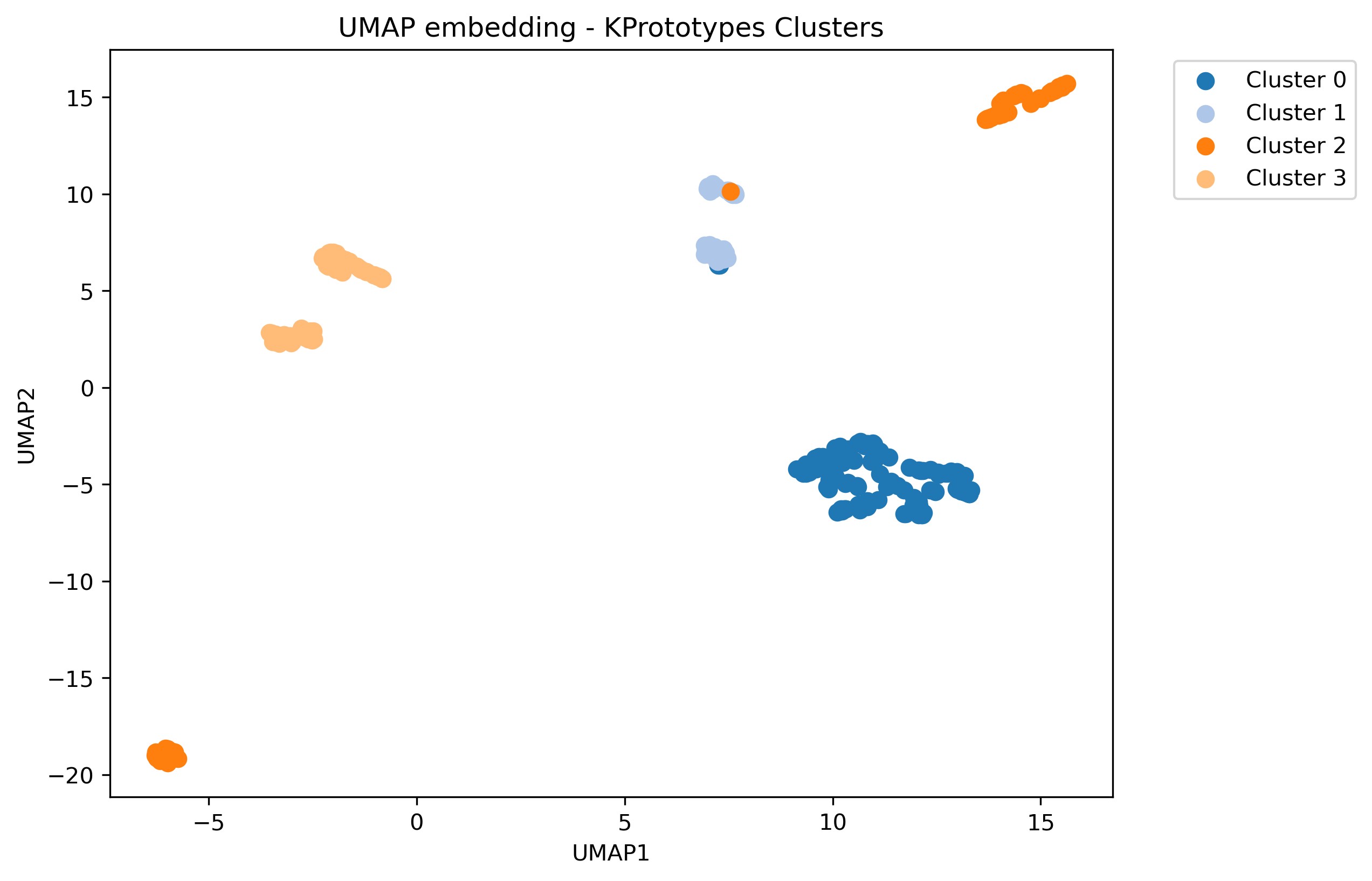

Supplement: SUPPLEMENTARY FIGURE S6 — UMAP plot showing the distribution of patients based on K-prototypes clustering. Each point represents a patient, colored by cluster. The plot illustrates cluster separation. [file Image_6.jpeg]
