# Supplementary material for: Unsupervised clustering of PET/CT features in fever of unknown origin (FUO) and inflammation of unknown origin (IUO)
Source: Front Med (Lausanne). 2026 May 29;13:1830800. doi: 10.3389/fmed.2026.1830800 (PMC13259882; doi:10.3389/fmed.2026.1830800)
Supplement: Supplementary file 7 [file Table_1.docx]

**Supplementary Table 1:** Distribution of Final Diagnoses According to FUO and IUO Status

|  | | **IUO (N=115, 39.8%)** | **FUO (N=174, 60.2%)** | **p-value** |
| --- | --- | --- | --- | --- |
| **Final diagnosis** | No diagnosis | 21 (18.3%) | 18 (13.5%) | 0.192 |
|  | Malignancy | 8 (7.0%) | 15 (8.6%) |  |
|  | Infection | 43 (37.4%) | 85 (48.9%) |  |
|  | Rheumatologic diseases | 30 (26.1%) | 38 (21.8%) |  |
|  | Other | 13 (11.3%) | 18 (10.3%) |  |
| **PET/CT involvement site** | Large artery involvement | 11 (9.6%) | 33 (19.1%) | 0.028 |
|  | Parenchymal involvement | 92 (80%) | 116 (66.7%) | 0.014 |
