# Supplementary material for: Unsupervised clustering of PET/CT features in fever of unknown origin (FUO) and inflammation of unknown origin (IUO)
Source: Front Med (Lausanne). 2026 May 29;13:1830800. doi: 10.3389/fmed.2026.1830800 (PMC13259882; doi:10.3389/fmed.2026.1830800)
Supplement: Supplementary file 8 [file Table_2.docx]

**Supplementary Table 2:** Comparison of PET/CT Involvement Patterns Between FUO and IUO Patients Using Multivariate Logistic Regression Analysis

|  | **IUO (N=115, 39.8%)** | **FUO (N=174, 60.2%)** | **Odds ratio** | **95% Confidence interval (95%)** | **p-value** |
| --- | --- | --- | --- | --- | --- |
| **Large artery involvement** | 11 (9.6%) | 33 (19.1%) | 2.2 | 1.055-4.58 | 0.028 |
| **Parenchymal involvement** | 92 (80%) | 116 (66.7%) | 0.5 | 0.286-0.875 | 0.014 |
