# Supplementary material for: Unsupervised clustering of PET/CT features in fever of unknown origin (FUO) and inflammation of unknown origin (IUO)
Source: Front Med (Lausanne). 2026 May 29;13:1830800. doi: 10.3389/fmed.2026.1830800 (PMC13259882; doi:10.3389/fmed.2026.1830800)
Supplement: Supplementary file 9 [file Table_3.docx]

**Supplementary Table 3:** Cluster Characteristics Based on PET/CT Features Using Gower Distance and Hierarchical Clustering

| **Clusters** | **Number of reticuloendothelial organs involved** | **Number of parenchymal organs involved** | **Number of large artery involved** | **Maximum SUVmax of the lymphoreticular system involvement** | **Maximum SUVmax of the parenchymal organ involvement** | **Maximum SUVmax of large artery involvement** | **Pattern of lymphoreticular system involvement** | **Pattern of parenchymal organ involvement** | **Pattern of large artery involvement** |
| --- | --- | --- | --- | --- | --- | --- | --- | --- | --- |
| **1** | 0.776119 | 0.0 | 0.0 | 3.623881 | 0.0 | 0.0 | No | No | No |
| **2** | 0.960674 | 1.780899 | 0.0 | 4.592753 | 7.097584 | 0.0 | Focal | Focal | No |
| **3** | 0.0 | 1.05 | 2.1 | 0.0 | 4.0595 | 5.146 | No | Focal | Diffuse |
| **4** | 1.583333 | 1.458333 | 1.75 | 6.806667 | 6.130417 | 5.209583 | Focal | Focal | Focal |
