# Supplementary material for: Unsupervised clustering of PET/CT features in fever of unknown origin (FUO) and inflammation of unknown origin (IUO)
Source: Front Med (Lausanne). 2026 May 29;13:1830800. doi: 10.3389/fmed.2026.1830800 (PMC13259882; doi:10.3389/fmed.2026.1830800)
Supplement: Supplementary file 10 [file Table_4.docx]

**Supplementary Table 4**: Distribution of final diagnoses across the four clusters derived from hierarchical clustering based on the Gower distance matrix of PET-CT features

| **Clusters** | **Infection** | **Malignity** | **None** | **Other** | **Rheumatologic_diseases** |
| --- | --- | --- | --- | --- | --- |
| **1** | 23 (34.3%) | 3 (4.5%) | 15 (22.4%) | 6 (9.0%) | 20 (29.9%) |
| **2** | 2 | 83 (46.6%) | 20 (11.2%) | 23 (12.9%) | 21 (11.8%) |
| **3** | 3 | 6 (30.0%) | 0 (0.0%) | 1 (5.0%) | 0 (0.0%) |
| **4** | 4 | 16 (66.7%) | 0 (0.0%) | 0 (0.0%) | 4 (16.7%) |
