# Supplementary material for: Unsupervised clustering of PET/CT features in fever of unknown origin (FUO) and inflammation of unknown origin (IUO)
Source: Front Med (Lausanne). 2026 May 29;13:1830800. doi: 10.3389/fmed.2026.1830800 (PMC13259882; doi:10.3389/fmed.2026.1830800)
Supplement: Supplementary file 12 [file Table_6.docx]

**Supplementary Table 6:** Distribution of final diagnoses across clusters derived from Gower distance–based HDBSCAN clustering. Cluster −1 represents noise (unassigned cases) as defined by the HDBSCAN algorithm.

| **Clusters** | **Infection** | **Malignity** | **None** | **Other** | **Rheumatologic_diseases** |
| --- | --- | --- | --- | --- | --- |
| **-1** | 29 (42.6%) | 6 (8.8%) | 8 (11.8%) | 6 (8.8%) | 19 (27.9%) |
| **0** | 9 (30.0%) | 0 (0.0%) | 9 (30.0%) | 2 (6.7%) | 10 (33.3%) |
| **1** | 14 (37.8%) | 3 (8.1%) | 6 (16.2%) | 4 (10.8%) | 10 (27.0%) |
| **2** | 27 (46.6%) | 4 (6.9%) | 11 (19.0%) | 4 (6.9%) | 12 (20.7%) |
| **3** | 29 (48.3%) | 6 (10.0%) | 2 (3.3%) | 12 (20.0%) | 11 (18.3%) |
| **4** | 20 (55.6%) | 4 (11.1%) | 3 (8.3%) | 3 (8.3%) | 6 (16.7%) |
