# Supplementary material for: Unsupervised clustering of PET/CT features in fever of unknown origin (FUO) and inflammation of unknown origin (IUO)
Source: Front Med (Lausanne). 2026 May 29;13:1830800. doi: 10.3389/fmed.2026.1830800 (PMC13259882; doi:10.3389/fmed.2026.1830800)
Supplement: Supplementary file 13 [file Table_7.docx]

**Supplementary Table 7:** Summary of PET-CT characteristics across the four clusters derived from spectral clustering of the FUO patient data.

| **Clusters** | **Number of reticuloendothelial organs involved** | **Number of parenchymal organs involved** | **Number of large artery involved** | **Maximum SUVmax of the lymphoreticular system involvement** | **Maximum SUVmax of the parenchymal organ involvement** | **Maximum SUVmax of large artery involvement** | **Pattern of lymphoreticular system involvement** | **Pattern of parenchymal organ involvement** | **Pattern of large artery involvement** |
| --- | --- | --- | --- | --- | --- | --- | --- | --- | --- |
| **0** | 1.42 | 1.87 | 0.00 | 6.81 | 7.63 | 0.00 | Focal | Focal | No |
| **1** | 0.00 | 1.60 | 0.00 | 0.00 | 6.00 | 0.00 | No | Focal | No |
| **2** | 0.86 | 1.27 | 1.91 | 3.71 | 5.19 | 5.18 | No | Focal | Focal |
| **3** | 0.78 | 0.00 | 0.00 | 3.62 | 0.00 | 0.00 | No | No | No |
