# Supplementary material for: Unsupervised clustering of PET/CT features in fever of unknown origin (FUO) and inflammation of unknown origin (IUO)
Source: Front Med (Lausanne). 2026 May 29;13:1830800. doi: 10.3389/fmed.2026.1830800 (PMC13259882; doi:10.3389/fmed.2026.1830800)
Supplement: Supplementary file 15 [file Table_9.docx]

**Supplementary Table 9:** Summary of PET-CT characteristics across the four clusters derived from K-prototype clustering of the FUO patient data.

| **Clusters** | **Number of reticuloendothelial organs involved** | **Number of parenchymal organs involved** | **Number of large artery involved** | **Maximum SUVmax of the lymphoreticular system involvement** | **Maximum SUVmax of the parenchymal organ involvement** | **Maximum SUVmax of large artery involvement** | **Pattern of lymphoreticular system involvement** | **Pattern of parenchymal organ involvement** | **Pattern of large artery involvement** |
| --- | --- | --- | --- | --- | --- | --- | --- | --- | --- |
| **0** | 0.64 | 0.47 | -0.37 | 0.57 | 0.38 | -0.37 | Focal | Focal | Diffuse |
| **1** | -0.03 | 0.02 | 2.24 | -0.11 | 0.01 | 2.23 | Mix | Focal | Focal |
| **2** | -0.28 | -1.17 | -0.37 | -0.33 | -0.86 | -0.36 | Mix | Diffuse | Diffuse |
| **3** | -1.05 | 0.29 | -0.39 | -0.79 | 0.14 | -0.38 | Mix | Focal | Diffuse |
