# Supplementary material for: Unsupervised clustering of PET/CT features in fever of unknown origin (FUO) and inflammation of unknown origin (IUO)
Source: Front Med (Lausanne). 2026 May 29;13:1830800. doi: 10.3389/fmed.2026.1830800 (PMC13259882; doi:10.3389/fmed.2026.1830800)
Supplement: Supplementary file 16 [file Table_10.docx]

**Supplementary Table 10:** Distribution of final diagnoses across clusters derived from K-prototypes analysis.

| **Clusters** | **Infection** | **Malignity** | **None** | **Other** | **Rheumatologic_diseases** |
| --- | --- | --- | --- | --- | --- |
| 0 | 56 (46.7%) | 16 (13.3%) | 12 (10.0%) | 17 (14.2%) | 19 (15.8%) |
| 1 | 27 (46.6%) | 4 (6.9%) | 11 (19.0%) | 4 (6.9%) | 12 (20.7%) |
| 2 | 22 (50.0%) | 0 (0.0%) | 1 (2.3%) | 4 (9.1%) | 17 (38.6%) |
| 3 | 23 (34.3%) | 3 (4.5%) | 15 (22.4%) | 6 (9.0%) | 20 (29.9%) |
